# Digital Narratives During the Pandemic: TV Series, Social Media, and Conversations on the Internet

**DOI:** 10.3389/fpsyg.2021.677713

**Published:** 2021-11-16

**Authors:** Rut Martínez-Borda, Julián de-la-Fuente, Pilar Lacasa

**Affiliations:** Department of Philology, Communication and Documentation, University of Alcalá, Alcalá de Henares, Spain

**Keywords:** TV series, digital storytelling, transmedia, big data, discourse analysis, content analysis

## Abstract

The situation of lockdown experienced during the months from March to June 2020 changed the daily lives of people in Spain and their leisure circumstances. This study analyses the narrative representations that people construct when they watched streaming TV series, during the covid-19 pandemic. To access these representations, the Spanish texts that appear on the Internet are analysed, including social networks and other social media. The paper adopts quantitative approaches that use big data analysis complemented with other qualitative approaches and inspired by content and discourse analysis. Findings show that these narrative representations constructed through conversations are on three levels in which context is revealed: first, institutional and community; second, online or offline interpersonal relationships which mention people as facts or as aspirations of their daily lives; and third, personal lives in the reconstruction of the series, projected on the plot reconstruction and the identity of the actors.

## Introduction

The pandemic has changed the lives of people a little. Social media has not been extraneous to these changes and neither have the representations people construct of its contents (Negra, 2020; Ong and Negra, 2020). Furthermore, we live in a world of multimodal universes, particularly visual ones, which are increasingly immersed in bodily experiences (Thurlow et al., 2020). Psychology cannot ignore these changes, considering how context affects the narrative that people construct in entertainment situations, for example, related to television streaming and expressed through social networks (Fiske, 1987; Stein, 2015; Lacasa, 2020). These representations are recent objects of study that psychology cannot renounce (Jewitt et al., 2016; Nærland, 2018; Parks, 2020). To analyse mental representations present through discourse is a particularly relevant approach to sociocultural psychology (Bruner, 1986, 2002; Demuth et al., 2020).

This situation also demands innovative methodological approaches which seek to integrate different perspectives in digital (Dencik, 2020). Big data, enabling new approaches to data, represent an alternative (Grimm et al., 2017, January; Woo et al., 2020) to traditional approaches that analyse quantitative questionnaires obtained in field studies or experimental work. In addition, when big data is obtained through social networks, its analysis can be complemented with other qualitative techniques, either content studies (Serafini and Reid, 2019) or discourse (Gee, 2014), which will provide a deeper contextualised meaning.

Against this backdrop, the questions stemming from this research study were defined as:

1. What narrative representations do people construct, related to certain television series, that they watch in streaming, in times of pandemic, and express through digital texts? How is the pandemic situation revealed in these representations and expressions?
2. What methodological challenges are posed by the analysis of conversations in digital environments, combining macro and micro levels? How can big data analytics be complemented by others that rely on content or discourse analysis to examine the situated meaning of narratives?

To answer these issues, this study approaches TV series during the pandemic. Big and small data analyses are combined to examine the digital texts around four television series, during the period between 14th March and 15th June 2020. This period coincides with the strict lockdown situation in Spain.

In our view, the main contribution of this paper is twofold. First, it shows that the representations that people build from the information they receive from the media need to be interpreted in particular contexts. Particularly, in this article, the TV series people watch in streaming. Second, from a methodological perspective, strategies are offered to combine analysis approaches related to big and small data.

The rest of this document offers, firstly, the theoretical framework of the study, secondly, the methodology used, and thirdly, the results and discussion raised by the research questions.

## Theoretical Framework

### Narrative, TV Series, and Situated Storytelling

Narrative has traditionally been the subject of psychology from multiple perspectives. Ryan (2010) relates it to cognitive psychology (Schaeffer, 2010) and experimental psychology (Stein and Trabasso, 1981). According to Bruner (1986, 2002) who represents a milestone from this perspective, the narrative is a way of thinking from which reality is interpreted. In this way, narrating is a way of constructing meanings. It cannot be separated from the culture or the discourse through which human beings communicate. Thus, stories are forms of narration that provide guides to speakers about how they can express themselves. In this paper, we delve into the stories that are built up from television. This requires an interdisciplinary approach in which socio-cultural psychology is enriched with other contributions, closely following the work of Ryan (2019), which explores narrative contexts related to digital communication.

Compared with the structural models of the traditional narrative, which tend towards abstraction by seeking universality (Barthes and Duisit, 1975; Genette, 1980; Greimas et al., 1990), situated storytelling imply forms of social interaction. Particularly, digital environments where personal expression is linked to dialogue (Ryan, 2005, 2007, 2019). This all happens within the framework of a community and the analyses must be supported in interdisciplinary frameworks which are linked to discourse analysis, to sociolinguistics, pragmatism, and especially psychology (Page and Thomas, 2011).

The TV series show stories that become situated storytelling, the meanings of which are generated in specific contexts such as social media. The social situation of those who are watching this TV series, impacts viewing and mobilises the significances which are specified into multiple representations. These may be expressed through discourse (Fiske, 1987; Hall, 1997). These digital and situated narratives are open, i.e., they have no final closure and are expressed through multimodal discourses (Bernhart and Urrows, 2019). Also, this trait may be understood in relation to the identity and personal presence in the narrative.

Preferences and motivations around a TV series involve individuals and communities (Hemphill et al., 2018). Added to this, relationships are established between online and offline contexts, because the narrative experience is an embodied experience. Our bodies are located in space, time, society, and culture. In other words, they are contingent to internal and external influences (Ensslin, 2011). This is what happens during the pandemic, when identity becomes an embodied reality in Internet (Boyd, 2008). Within this framework, the concept of participative discourses becomes highly relevant, where stories are built up from the contents of the media (Thornborrow, 2015). Narration is understood as an activity related to discourse within a framework of a participative culture where implicit and explicit norms are imposed. These help to shape the community and encourage the coexistence between experts and followers (Demuth et al., 2020).

### Transmedia Television Stories

Referring to TV series, transmedia stories must be understood within the framework of the changes made by Internet and digital platforms in the media context. New ways of viewing series arise, summarised by the maxim of anywhere, anytime, any device (Combes and Glevarec, 2020). The traditional television and, with it, the shows which were introduced in the form of soap operas (Piñón, 2019) have been forced to redefine their nature, triggered by technological changes.

Probing into digital stories leads to talk of transmedia, a perspective which is based on dynamic storytelling (AnsGar, 2003). Furthermore, transmedia storytelling has been linked to a combination of tools which lead to prototypical aspects of the storytelling through new media and to those considered to be engagement strategies (Mittell, 2015; Thon, 2016).

Relationships between *transmedia narratology* and *transmedia storytelling* (Ryan, 1991, 2016; Passalacqua and Pianzola, 2011) are established beyond media. The term is becoming ever more popular through the works of Jenkins et al. (2006, 2017), and Jenkins (2010), in relation to contemporary culture. The media, which are the nuclei of the transmedia phenomenon are somewhat more than information channels, they are also a means of artistic expression.

The concept of transmedia has aroused interesting debates on the way in which people represent histories in the media: a good example of this is the debate published in the International Journal of Communication (Kavoori et al., 2017). This includes interviews with representatives of narratology, where they reflect and debate the transmedia concept. It is worth pointing out the two positions here due to their relevance in this study. The first, insists that the construction of the transmedia stories are a process involving the participation of the audience to the contents offered by the media (Papacharissi et al., 2017). Many voices interact in the construction of texts, behind each of which they are able to hide their different identities and situations (Archakis and Tzanne, 2005; Larrain and Haye, 2019). The second, related to the viewpoint of Murray et al. (2017), is highly critical towards the version of the transmedia as a participatory narrative.

### Interactive and Multimodal Stories

This study analysed how the stories were reconstructed through social media, using interaction processes between users, texts, and platforms. These social networks are the framework in which we contextualised the interactivity. Compared with traditional media they are characterised by three traits. First, they include content generated by users; second, they provide social interaction tools; third, they engage in commitment which are generate collaborations, multiple modes of participation and communities with shared values and goals (Chung and Yoo, 2008; Sloan and Quan-Haase, 2017).

Other studies (Page, 2010, 2012) have shown that, in this digital environment, stories are fragmented. Sometimes, they appear from other discourses and are outside of what has traditionally been considered storytelling. Researchers linked to linguistics, psychology, or socio-cultural anthropology refer through discourse to these disperse imaginary or figurative worlds (Holland et al., 1998), since the words make sense within the framework of the stories. These are storyworlds which are built up collectively, from the community framework (Page, 2012; Nærland, 2018; Sundet and Peteresen, 2020).

These discourses, developed through social networks, and from which imaginary worlds are collectively constructed were characterised by two traits in the works by Marie Laure Ryan: interactivity and multimodality (Ryan et al., 2017). Interactivity (Ryan, 2011, 2015) refers to digital stories using a metaphor. Particularly, the presence of different layers: (a) peripheral interactivity. The text maintains a unit of form and content at an interface level. In fact, it is a combination of fragmented stories; (b) interactivity affects the narrative discourse and not the presentation of the story. The materials are predetermined but presentation is dynamic; (c) interactivity creates variations in the predefined story. The user forms part of the world of the story and provides freedom of action. This is typical of videogames; and (d) the generation of the story in real time. At this level, the stories are not predetermined, but they are generated by data that come partly from the system and partly from the user. It may become clear that the layers follow a logical order from lesser to greater interactivity. From stage four, we try to find a meaning by analysing our data. Here, the concept of possible worlds begins to make sense (Ryan, 1991, 2006) because they are the representations which the people build from the story, as part of interactive systems.

The second concept proposed by Ryan et al. (2017) is that of multimodality, which occupies an essential place in communication through the computer (Page, 2010, 2012). Multimodality is also related to the theoretical models of socio-cultural psychology (Kress and Van Leeuwen, 2006; Kress, 2012). Modes are semiotic resources from which the significance is constructed and are highly varied. These resources may be gestures, sounds, images, or words (Jewitt et al., 2020; Thurlow et al., 2020). As for the construction of narratives, this implies that they are using many resources, beyond oral or written language, in any communicative setting. Participants in social networks create significances collectively using multimodal semiotic resources. Social semiotics is particularly of interest (Jewitt et al., 2016, 2020) because it emphasises the activity (agency) of the people who construct the significance in social contexts. Moreover, the systems are built up from social usage, and this is what happens when representations and imaginary worlds are created through social networks or other digital texts.

## Methodological Approach

This project is based on the quantitative analysis of “big data,” which are defined by four properties: volume, since they include terabytes or petabytes; speed, because they are obtained in real time and space; variety, in that they can be structured or unstructured; and comprehensiveness in the objective, since they capture the entire population and are generated continuously (Zikopoulos, 2012; Kitchin, 2014; Panda et al., 2018). In addition, qualitative analyses are carried out from the perspective of content analysis (Gee, 2014; Jones et al., 2015; Tannen et al., 2015; Zamith and Lewis, 2015; Armborst, 2017; Recuber, 2017). It is, therefore, a methodology in which complementary approximations are used (Paoletti et al., 2021).

This research analyses the construction of digital stories relating to a selection of television series interacting with mobile devices. Digital texts, related to TV series, were collected during the period between 14th March and 15th June 2020. This period coincided with the situation of strict lockdown in Spain. For this we have used a specific software big data analysis. Conversational analysis software that performs content are based on two possible strategies, which are used interchangeably across different analysis processes: (1) the presence and absence of certain linguistic terms; (2) the semantic meaning generated by the software. It should be noted that the software also allows access to the original source at any time, something that makes it easier for the researcher to go beyond big data and carry out qualitative discourse analysis processes that seek meaning through interpretations.

### The Selected Series

To select the series, a questionnaire was created through a Google template, to which 110 young people aged between 18 and 23 responded. The questionnaire was conducted in the months of January and February 2020 before the unforeseen confinement occurred which, in Spain, began on March 14, 2020. The purpose was to find out which series interested young people more and the reasons of their preferences. There was a huge variety of answers. From the most mentioned series we selected those that led to contrasting interpretations regarding youth themes and others aimed at wider audiences. Also, two were Spanish and two British. These were the four series around which conversations were analysed:

• “Money Heist” (2017, Spain). Broadcast on Netflix, the fourth season began on April 3rd, 2020, during the period analysed. The plot revolves around two heists in emblematic places in Spain: *Casa de la Moneda* (the Mint) and *Banco de España* (the Bank of Spain).
• “Elite” (2018, Spain). A youth themed series, broadcast in 190 countries. It narrates the life of a group of students in an elite school, where different social classes coexist due to the grant system for some of them. The third season began on 1st March 2020.
• “Sex Education” (2019, United Kingdom). The plot is related to an insecure young man who has an answer to anything related with sex because his mother is a sexologist. A classmate of his school encourages him to open a sex consultation service. The second season began on 17th January 2020.
• “Peaky Blinders” (2013, United Kingdom, BBC). Five seasons, the last began on 22nd September 2019. A family of gangsters from Birmingham after the First World War (1914–1918), control a horse racing betting establishment. It covers adventures and conflicts with the police.

### Analysis Processes

The first challenge faced by the researcher is to channel the flow of digital texts. The minimum units are what is defined as mentions. Particularly, any message or publication filtered from the terms of the titles of the series collected on Twitter, YouTube, Facebook, and digital texts that appear on the internet (for example, news, forums, or blogs). Each of these mentions are issued by certain users, which can be accessed directly through their personal or institutional accounts. Each one can be the author of one or multiple mentions. The software provides demographic data, such as age or gender, and most prominent users—those who have sent more messages who have had greater impact due to the number of their followers. We should emphasise that this type of study includes the total population that participated in the social network within the specified data input period. This is not a representative sample.

**FIGURE 1:**
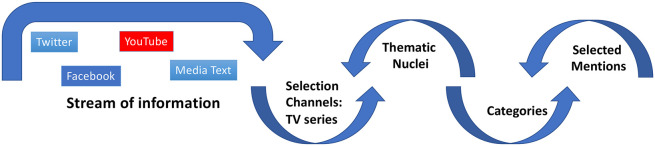
Analysis processes.

We combined quantitative and qualitative analyses. A synthesis of the analysis processes used in contained Figure 1. Information seeps progressively through circular analysis in which these processes interact.

First, define channelling information from the terms used in the indications. These channellers are relevant for answering the research questions (Kitchin, 2014) and, in the case of this study, four were used, each of them corresponding to the series viewed.

Second, define a set of core themes, semantic, or conceptual units, which arise from the terms most frequently mentioned through a process of content analysis conducted by theoretical assumptions related to the research objectives.

Third, the categories will be grouped into these nuclei. This is dynamic since they can vary and adjust whilst the flow of the conversation lasts (Panda et al., 2018). To define the system the presence of certain terms as the theoretical model on which the work is based will be considered.

Fourth, each of the mentions can be analysed from the perspective of discourse analysis, contextualising it in the flow of specific conversations in order to define the meaning that the speaker seems to attribute to it (Gee, 2014; Jones et al., 2015).

These processes interact circularly over the course of input and data collection. They have conditioned the way this work presents data sources, analyses, and results. Finally, it should be noted that several transcripts exemplify the thematic nuclei of the analysis. The reason for selecting these and not others is a decision of the research, considering them as relevant examples of the category. Discourse analysts (Gee, 2014) point out the limitation related to the fact that not all transcripts could be analysed in-depth. It should note that the software allows access at any time to the original text and reviewing in deep as necessary. It is up to the researcher to choose the examples to consider. The decision relates to the degree of representativeness of the transcript, in the opinion of the investigator, about the category exemplified.

## Channelling Relevant Information: Sources of Information, Evolution, and Demography

### Sources

Observing the sources from which data are generated, differences appear regarding the weight of each of these sources. Of the total mentions (17,735), those with a greater weight correspond to digital texts, called media (6,594), and Twitter (6,408). The weight of each source in each of the series was considered and appears in the table.

Considering the weight of each medium in the total mentions of each series, two patterns are observed in Table 1. The British series in which the mentions that appear on Twitter have a greater weight (Sex Education, 77%) and Peaky Blinders (84%). The fact that it is in the Spanish series (Money Heist, 42% and Elite, 56%) where the media acquire a greater weight, may be because expert criticisms appear in digital magazines more than in social networks.

**TABLE 1:** Sources, frequencies, and percentages.

	**Money Heist**	**Elite**	**Sex Education**	**Peaky Blinders**	**Total**
Media	3,776 (42%)	2,394 (56%)	151 (22%)	282 (16%)	6,594
Twitter	2,837 (31%)	1,574 (37%)	526 (77%)	1,471 (84%)	6,408
YouTube	1,673 (19%)	315 (7%)	6 (1%)	3 (0%)	1,997
Facebook	715 (8%)	21 (0%)	0 (0%)	0 (0%)	736
Total	8,992 (100%)	4,304 (100%)	638 (100%)	1,576 (100%)	15,735

### TV Series During Lockdown

An initial observation of the data show that the mentions of the series during total lockdown, when people were unable to go out of their homes, account for 87% (13,695 mentions) of those analysed over 3 months. These data may be related to others which show how Internet use increased in homes during the strictest stage of lockdown (Belson, 2020, May 28).

**FIGURE 2:**
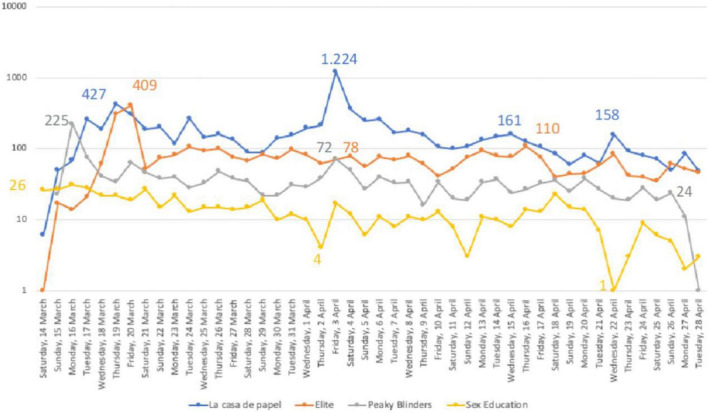
Semi—logarithmic scale. Evolution of the references to coronavirus in the first lockdown phase.

Figure 2 contains the evolution of the number of mentions during this initial stage of total lockdown, through a semi-logarithmic scale, whereby the evolution of the number of mentions in each series may be compared.

The data show different patterns regarding the number of mentions broadcast. The most relevant data is that which occurred on 3rd April in relation to Money Heist (1,224 mentions), when the fourth season began. It also highlights the fact that the other Spanish series, Elite, which deals with youth themes, saw a progressive rise until 20th March, up to 409 mentions, which then dropped to 46 the following day and hardly varied over time. A relatively inverse role occurred with Sex Education, where 2nd April was one of the lowest days. Peaky Blinders, the series with a more continuous profile, had its highest peak on 15th March (225 mentions) and this oscillated until the second peak (73 mentions), which then stabilised until almost the end in a continuous line. These data show that, apart from lockdown as such, there are other factors arising from context, especially at the beginning of a season.

### Users

Table 2 includes the total data obtained and considered, monitored through the Coronavirus^1^ filter. Both frequencies and percentages were included. As may be observed, the number of mentions was presented and the users who procured them, together with the sources which generated them.

**TABLE 2:** Mentions and users.

	**Money Heist**	**Elite**	**Sex Education**	**Peaky Blinders**	**Total**
Mentions	8,992 (57%)	4,304 (27%)	683 (4%)	1,756 (11%)	15,735 (100%)
Users	3,977 (53%)	1,703 (23%)	495 (7%)	1,377 (18%)	7,552 (100%)

Regarding mentions, there was a total of 15,735 mentions by 7,552 users, although distribution implied notable differences between the series. Money Heist, where mentions account for 57% of the total (8,992 mentions) is outstanding, compared with the 27% (4,304, mentions) for Élite, and the much lower percentages for Peaky Blinders (11%, 1,756 mentions) and Sex Education (4%; 683 mentions). A relatively similar distribution was observed regarding user percentages. Specifically, 53% gave mentions (3,977) in Money Heist, 23% in Elite (1,703 users), 18% in Peaky Blinders (1,377 users), and 7% in Sex Education (495 users).

These data show that the Spanish series, Money Heist and Elite generated higher audience participation. It is relevant that Elite began its third season on 13th March and Money Heist on 3rd April, which no doubt generated a higher volume of mentions in conversation. In both cases, the contents of the corresponding series were available the day they began to be streamed through Netflix.

Demographic data are scarce, since users do not always offer this information. Regarding gender, of the 4,580 users, 2,511 were men (55%) and 2,069 were women (45%). There were no differences between the series. Regarding age, only 307 users offered this data, with most (177 users, 58% of the total) being aged between 18 and 24 years of age; 56 personas stated they were aged between 24 and 30 (18%); and 31 users (20%) being under 18; 43 people (14%) were over 30 years of age.

## Searching for Meaning Towards Thematic Nuclei

**FIGURE 3:**
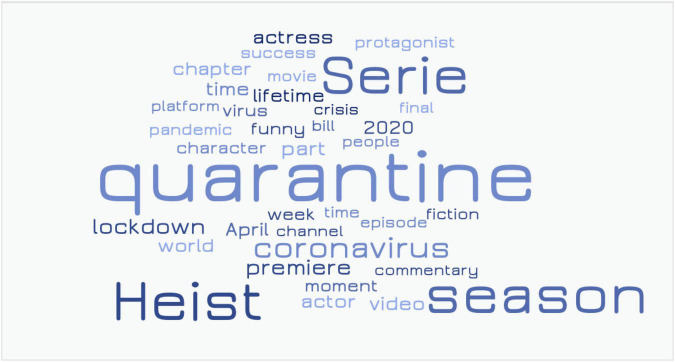
Word cloud. Terms used MOST OFTEN in conversations.

In this study, we examine the content of conversations on the Internet, to analyse the representations that users construct of the TV series they watch. This requires looking for the semantic proximity of the terms used in these conversations (Armborst, 2017). The data related to the content of these conversations arise from the analysis of the most frequent terms in the mentions of the series, included in Table 3 and Figure 3.

**TABLE 3:** Frequency of the most used terms in conversations.

**Terms**	**Frequency**	**Terms**	**Frequency**	**Terms**	**Frequency**	**Terms**	**Frequency**
Quarantine	5,628	Video	669	Funny	454	Commentary	297
Money	4,508	Time	629	Virus	445	Final	291
Heist	3,907	Actor	603	Week	416	Episode	284
Season	3,789	Chapter	553	Movie	402	People	276
Serie	3,764	2020	537	Pandemic	365	Bill	273
Coronavirus	1,592	World	529	Success	353	Channel	272
Premiere	879	Lifetime	508	Time	352	Crisis	269
Lockdown	859	April	505	Protagonist	337		
Part	685	Moment	490	Fiction	323		
Actress	669	Character	477	Platform	308	Total:	37.497

Our methodological challenge requires combining quantitative and qualitative approaches (Paoletti et al., 2021). This also implies an interaction between theory and data through a double process that is inductive (most frequent terms considered as shown in Figure 3) and deductive, because to define the thematic nuclei that group the categories, the theoretical model on which the research questions are based is also considered (Boellstorff, 2012).

In consideration of the semantic meaning of the terms appearing in Figure 3, three thematic nuclei were defined for the organisation of the different categories.

*Coronavirus setting*: this includes terms such as quarantine, covid-19, or lockdown. They allude to the general context of the pandemic in which the series was viewed and allowed us to talk about situated narratives (Page and Thomas, 2011; Ryan, 2019).

*Immediate contexts*: everyday routines and social relationships. Terms such as week, people, and lifetime are included (Page, 2010; Sundet and Peteresen, 2020).

*Multiple worlds figuring in the series*: the presence of terms such as lifeworld, protagonist, fiction, actress, or character.

**FIGURE 4:**
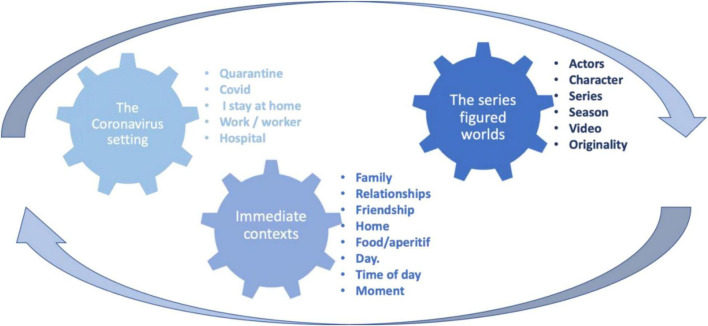
Thematic nuclei and categories.

Figure 4 shows these nuclei along with the categories that each one includes. All of this will allow us to delve into the representations of the series that people have built in times of the pandemic. They are progressively defined combining quantitative and qualitative analysis.

## TV Series During Coronavirus

### Coronavirus Setting

The first thematic core directly refers to the lockdown situation in which people watch the series. The categories included in this nucleus appear in Table 4. Its definition is related to the terms from which the category has been delimited in the search in Spanish.

**TABLE 4:** Analysis categories related to the coronavirus setting.

**Categories**	**Terms and concepts included in the search in Spanish**
Quarantine	Quarantine, confined (singular), confinement, locked down (plural), locked down (singular), lockdown, confined (plural).
Covid	Covid, covid19, covid2019, covid-19, covid-2019, covid_19, covid_2019, cvd.
Stay at home	I am staying at home, I stay at home, Iamstayingathome, Istayathome, stay at home, stayathome, stayingathome athome.
Work/worker	Work, Works, male worker, female worker, workers, occupation, job, labour, labours, we work, I worked, they worked, he worked hard, they worked hard, fight, they fought, I fought, he fought, work hard, getting on with it, laborious, diligent, hard-working, entrepreneur, solicitous, hard-working, untiring, indefatigable, industrious, blue-collar worker, operator, day labourer, salaried, producer.
Hospital	Medical centres, clinic, clinics, hospital, hospitals, outpatients, healthcare, healthcare professional, ambulance worker.

The data included in Table 5 show that the category with the most weight is, in all cases, the one related to references to covid. It is higher in the case of Peaky Blinders (80%) and somewhat lower in Elite (67%). They also allude, in second place, to quarantine, as it is distributed between 20% in Elite and 52% in Sex Education. These data show that the phenomena directly related to the pandemic are more relevant than the consequences that derive from it, such as hospitalisation and even the fact of necessarily staying at home (Werron and Ringel, 2020).

**TABLE 5:** Frequencies and percentages of mentions regarding the coronavirus setting.

	**Money Heist**	**Elite**	**Sex Education**	**Peaky Blinders**
Quarantine	1,117 (17%)	628 (20%)	52 (9%)	174 12%)
Covid	4,852 (73%)	2.062 (67%)	442 (77%)	1,129 (80%)
I stay at home	205 (3%)	204 (7%)	16 (3%)	15 (1%)
Work/worker	159 (2%)	52 (3%)	11 (2%)	30 (2%)
Hospital	297 (4%)	142 (2%)	53 (9%)	60 (4%)
Total	6,630 (100%)	3,088 (100%)	574 (100%)	1,408 (100%)

Through a qualitative analysis, it is possible to delve into the meaning that people attribute to their practices. Transcription 1 contains a set of examples where discourse analysis complements the big data. These mentions which appear on Twitter reveal that the participants express situated narratives, talking from many voices and viewpoints, set in time. There are dynamic texts where the people reinterpret the series content (Page and Thomas, 2011; Ryan, 2019) and, as observed in the texts, talk about aspects which are closely linked to their own circumstances, and to those the series has represented at that time of the lockdown.

**Transcript 1^2^. Series as forms of escapism during lockdown.**

The beginning 14–15th March

*1.1. Pakito OD @pacoliaco (11:44 PM ⋅ Mar 15, 2020)*
I’ve just finished day two of being locked inside the bedroom. I have watched 3 seasons of Elite in two days. Now what can I watch? Any recommendations? When does the new @lacasadepapel season start??? @NetflixE you could bring out new cool things.
https://twitter.com/pacoliaco/status/123932172455186 0227
*1.2. Suelta La Sop @SueltaLaSopaTV (7:00 PM ⋅ Apr 27, 2020)*
The main character of Money Heist, is living in a nightmare together with her husband and experiences weird things in her body after testing positive for coronavirus.
https://trib.al/MyotSlz
2 Likes
https://twitter.com/SueltaLaSopaTV/status/12548176273 45768448
The concept of the situated and interactive storytelling implies a set of meanings (Fiske, 1987) which audiences express in relation to the situation the pandemic has put them in. The meanings are related to the voices that express them. Statements in the first person appear which express feelings or emotions generated by the situation, “Well, I have just finished day two of being shut in my bedroom” (1.1), that reveal the awareness of the first day of lockdown.

A different voice, which to some extent, could be that of the narrator is introduced in the fragment 1.2. ‘‘The main character of Money Heist, is living in a nightmare together with her husband and experiences weird things in her body after testing positive for coronavirus.’’ Looking at the sending of the message, in this case, it is not a fan but a series follower. The author, @SueltaLaSopaTV, is from an entertainment portal TeleMundo,^3^ a north American TV station, which communicates in Spanish, with accounts on Integra and Facebook. It may be said that whoever issues this type of message works within a participative culture (Thornborrow, 2015) and tries to take on the role of someone expert who speaks in the third person. The texts are produced in a digital environment to allow the digital media to talk to the audiences and impact the way in which contents are generated and interpreted (Rudrum, 2005; Monfort, 2007).

### Immediate Contexts: Everyday Routines and Social Relationships

Below is the content of messages grouped around a system of categories thematically linked to the relevant context relating to Coronavirus. The social relationships mentioned by the message issuers appear (family, relationships, friendship). Spatial situation, where the home or accommodation is referred to and the time period, alluding to different stages of the day and to specific moments. These categories are continued in Table 6.

**TABLE 6:** Analysis categories related to close context.

**Categories**	**Terms and concepts included in the search**
Family	Family, relative, families, relatives, family, familiarity, familiarise, familiarised, multifamiliar, unifamiliar.
Relationships	Relation, relationship, relations, partner, partners, male friend, female friend, male friends, female friends, family, familiar, families, married couple.
Friendship	Friends, colleagues, team, friendship, female friends, female friend, friends, loyal friend, loyal friends, loyalty, friend,
Home	House, flat, suite, home, detached house, attic flat, houses, flats, my room.
Food/aperitif	Aperitif, aperitifs, snack, snack, bite, snacking, fridge, eats tapa, tapas, snackette, snackettes, eating between meals, snacking, to snack, canapé, eats, refreshments, canapés, canape, canapes, roll, morsel, bite to eat, bag of crisps, nachos, popcorn, cheese balls, risquetos, sunflower seeds, mention or appraisal of food as a product, food, meals, to eat, eating.
Day.	Days, days, days.
Time of day	Morning, mornings, first thing morning, morrow, night, nights, early morning, early mornings, evenings, afternoon.
Moment	Moment, moments, instant, instants, now.

In this case, Table 7 shows the patterns regarding category distribution, which in the series are similar with the exception of Elite where references to food or aperitif are practically absent (0.30%). It is of note that the weightier categories are social relations (e.g., Money Heist, 1980 mentions, 21%) and references to specific moments (e.g., Peaky Blinders, 266 mentions, 24%). Here are some examples:

**TABLE 7:** Frequencies and percentages of mentions regarding close context.

	**Money Heist**	**Elite**	**Sex Education**	**Peaky Blinders**
Family	507 (5%)	364 (6%)	48 (8%)	67 (6%)
Relations	1,980 (21%)	1,386 (24%)	105 (17%)	167 (15%)
Friendship	1,620 (17%)	1,199 (21%)	103 (16%)	160 (14%)
House	644 (7%)	518 (9%)	45 (7%)	78 (7%)
Food moment	1,559 (16%)	17 (0,30%)	127 (20%)	219 (20%)
Day	768 (8%)	532 (9%)	52 (8%)	93 (8%)
Day time	670 (7%)	498 (9%)	19 (3%)	71 (6%)
Moment	1,826 (19%)	1,148 (20%)	131 (21%)	266 (24%)
Total	9,574 (100%)	5,662 (100%)	630 (100%)	1,121 (100%)

**Transcript 2. Time and space**

**Meal**’**s time**
*2.1. @marianietodiaz (24 mar 2020–9:00 a.m.)*
Being in lockdown has led to new routines in my life. One of the ones I most enjoy: having breakfast whilst watching a chapter of a series. Last week it was #Élite, this week, #VidaPerfecta.
645 Following–1,776 Followers
1 like
https://twitter.com/marianietodiaz/status/124236069599 7575168
**Home**
*2.2. Highlightedcomment (8 months ago)*
Comment in the Élite account | Advert of season 4 Youtube
Diana Rodriguez
It’s funny the way these news items fill our hearts during this pandemic, the guys seem to speak to each one of us at home. Watching them both peaceful and excited at the same time is contagious and waiting to go out becomes more light-hearted. Thanks to you!!
https://www.youtube.com/watch?v = LM7aicp70UA and lc = Ugw1MczpbX5_7aGnUiB4AaABAg
**Day**
*2.3. @MariaPalmero_ (10:17 a. m.–3 abr. 2020)*
It’s Friday already. What is bad? That we continue to be shut in. What is good? That we are still alive. The best? That this evening I have plans: see #LCDP4 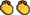 (I would prefer to go dancing but everyone has to get in a good mood any way they can my friends.) # Happy Friday and I really recommend the series @lacasadepapel https://t.co/ssUr57ejLS
11 likes
1,138 Following 8,547 Followers
https://twitter.com/MariaPalmero_/status/124598881306 0689921.

Audience practices are relevant and associated with watching series (Transcript 2.1). As a regular and pleasurable habit, mealtime helps to organise life in the pandemic to some extent (Werron and Ringel, 2020). One could also think that the reference to the home, to one’s own house, could be agreeable but it is not always the case. On the one hand, the house alludes to a lockdown situation: *“the guys seem to speak to each one of us at home. And waiting to go out becomes more light-hearted”* (Transcript 2.2). In this case, the fragment is a comment about one of the videos which appear on the YouTube account of the series, Elite, which they have highlighted as the most relevant. The message has been generated in a transmedia context (Jenkins et al., 2017; Sundet and Peteresen, 2020).

Let us now look at the time references, another dimension of the context. Those who issue the message are in immediate time, related to watching the series and to other types of daily life activities (Lacasa, 2020). When fragment 2.3 is examined in detail, the author is defined in the profile as a journalist with 8,547 followers. People build up stories (Archakis and Tzanne, 2005) relating to their daily life, meaning that the TV series becomes partly their own story. Again, it is a transmedia context (Passalacqua and Pianzola, 2011), because in the text, social media are combined with a series which they will have surely watched from a streaming platform. We can clearly see that these media contents define leisure time during the pandemic.

Below, in Transcription 3, there is another of the dimensions which defines the context, close or distant social relations. Two relevant themes have been selected: family and looking for a partner.

**Transcript 3. Social relations**

***The family***
*3.1. @esunen (16 mar. 2020/9:58 p. m)*
My daughter aged 16 has suggested we watch a series together to help pass lockdown. Obviously we said yes and we accepted that she choose it, so I have been watching Sex Education for ten minutes. this lockdown is going to be very long.
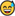
879 Following–5,283 Followers
28 Retweets/4 Tweets citados/299 Me gusta
https://twitter.com/esunen/status/1239657225523802112
***Looking for a partner?***
*3.2. neofjcn01 (2020/04/13–17:06)*
am using Tinder these days in lockdown to meet people. I ask them for recommendations on series. You can’t imagine how many people have recommended this rubbish to me. I immediately block them for their basic and vulgar tastes. Greetings.
Comment on YouTube
Highlighted comment
https://www.youtube.com/watch?v = SIWDVc145zU and lc = Ugzq5gcJqFsChYuNjtR4AaABAg.

We see that the texts relate to family. Again, comments on the series associated with certain practices appear (Lacasa, 2020). In fragment 3.1, the series is linked to a way of passing the time in lockdown: *“My daughter aged 16 has suggested we watch a series together to help pass lockdown.”* The author, with 5,283 followers, whose profile says is a politician, is talking in the first person. He expressed ambiguous feelings. Particularly, they seem to be happy to watch *Sex Education* with the daughter but accept that the situation is a bit strange.

Fragment 3.2 also appeals to the need to establish social relations. Again, it places us in a transmedia context (Stein, 2015) which introduces personal stories. In this case, when someone tries to find a partner. It is a YouTube comment, to one of the videos from that application in Money Heist and appears as a highlighted comment. The issuer of the message mentions in their comment one of the mobile apps they usually use to look for new dates, Tinder. They ask for recommendations for passing the time but reject the answers of those who respond. The message generated two interesting comments which reproach the author for not respecting the opinions of others and for depreciating the tastes of others because they are deemed vulgar.

### Multiple Worlds Figured in the Series

From the fan community, collective ideas of the series are built up, organised in figured worlds, especially around the actors. Also, the content structure, which is presented through successive seasons, crosses over with continuous views in streaming through different platforms. This is what the categories of Table 8 show, which refer to the content and form of the series.

**TABLE 8:** Analysis categories related to the series content and form.

**Categories**	**Terms and concepts included in the search**
Actors	Actors, cast, actor, actress, actresses, role, roles, artist, artists, staff.
Character	Characters, character.
Series	Mention or appraisal of the contents of the televised broadcasting analysed when they are serials, a series or several series.
Season	Season, part, parts, seasons.
Video	Vídeo, video, vídeos, videos, clip, clips, streaming.
Originality	Originality, original, interesting, unique, unique (plural), odd, astounds, interests, interested, amazing, new, unparalleled, exemplary.

Table 9 includes a summary of the big data obtained in relation to these categories.

**TABLE 9:** Frequencies and percentages of the mentions regarding form and content.

	**Money Heist**	**Elite**	**Sex Education**	**Peaky Blinders**
Actors	2,421 (16%)	1,098 (13%)	102 (10%)	142 (9%)
Character	1,443 (9%)	649 (8%)	93 (9%)	112 (7%)
Originality	2,025 (13%)	1,470 (17%)	118 (11%)	201 (13%)
Series	4,226 (27%)	2,502 (29%)	389 (37%)	594 (38%)
Season	3,602 (23%)	1,905 (22%)	265 (25%)	434 (28%)
Video	1,679 (11%)	875 (10%)	87 (8%)	88 (6%)
Total	15,396 (100%)	8,499 (100%)	1,054 (100%)	1,571 (100%)

Let us first look at the big data. Again, the patterns are similar. For example, the category “series” is the one with the greatest weight, particularly in Sex Education (389 mentions, 37%) and Peaky Blinders (594 mentions, 38%). Regarding references to actors, patterns are also like those of Money Heist (2,421 mentions, 16%) and Elite (1,098 mentions, 13%).

Several examples are cited below. Interactivity may be observed between cultural industries and audiences in creating variations in the predefined history. We recall the contributions of Ryan (2011, 2015), who proposed the construction of digital stories into several layers.

**Transcript 4. The plot**

*4.1. YouTube 11 THINGS P5 of MONEY HEIST has to resolve | Netflix España. 1.16M subscribers* (May 29, 2020)
284,073 views
Like 11K.110 Unlike
Netflix España 1.16M subscribers
What will happen to Gandía? and Little Arthur?What will Alicia do with El Profesor? How will they get the gold out of the Bank of Spain? There are 11 things from part 5 here from Money Heist which need answers.
#PreguntasNetflix #LCDP #NetflixEspaña
Patricio Pulido 8 months ago
I think Palermo will sacrifice herself or die saving the band, just as Berlin did in season two.
636 like. 26 replies
https://www.youtube.com/watch?v = pNkdkF5Gtf4 and lc = Ugwl6hR4eNrx6rhfqiR4AaABAg-
*4.2. Unknown (24 march 2020–15:47 h)*
I already know how this great series will end. They will all die of COVID except those who are inside the bank because nobody was contagious and they were in lockdown (but in the bank) the professor will be dying but because he trained the others how to operate and know things like doctors, they will save the professor’s life. And Spain will begin from 0 but the attackers will start a new life and without any risk of being captured, Río and Tokyo will be in charge of bringing new generations into the world.
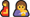
https://www.facebook.com/lacasadepapelnetflix/posts/11 53528771664255?comment_id = 690473818374026.

The fragments contained in Transcription 4 show that management is very different depending on the message sender, i.e., informative headlines or individual users. For instance, fragment 4.1 is sent from the official account of Netflix on YouTube. After the fourth season, the title is highly relevant: “*11 THINGS which P*5 of *MONEY HEIST has to resolve | Netflix España*,” and after this, a set of questions are put forward, relating to the plot but seen from the actions of the characters. The idea is to trigger a dialogue through an interactive process with the fans (Lacasa, 2020). If you go to YouTube and look at the comments, the producers have achieved what they appeared to desire. It says, e.g., *“I think Palermo will sacrifice herself or die saving the band, just as Berlin did in season two.”* This comment, in turn, generated a discussion within the community. It is obvious that the producer is directing and monitoring the interventions in social networks in his or her interests, e.g., towards where the next season could go (Page, 2012).

Focusing on fragment 4.2, we observe that the author of the message is reconstructing the plot of the future season of the series, in keeping with their wishes, which are no doubt conditioned by the pandemic as evidenced by the quote, *“They will all die of COVID except those who are inside the bank because nobody was contagious, and they were in lockdown.”*

As shown, the characters and actors play a relevant role in the story plot in generating content, and this is what the fragments of transcription 5 demonstrate.

**Transcript 5. The characters and the plot**

5*.1. Ricardo joseGuarin gonzalez (10 months ago (edited))*
Highlighted comment in MenteVáhez
https://www.youtube.com/channel/UCBj-xjl23MHAwH4SRD3Ytvg
I don’t think Alicia joins the band, it would be disappointing to lose the best enemy. Lisbon comes in to replace Palermo as the leader that is for definite. Now Palermo will be more important than anyone not because he is willing to sacrifice himself for his feelings of guilt, but for his commitment to the success of the heist. I don’t think they will give away the gold and get confused with their followers. It would be too obvious and predictable. They will get the gold out and use it in exchange for their “freedom,” using power and social legitimacy acquired from demonstrating the tortures and unlawful government procedures. The vital thing is no longer the money. But I also know that none of this will happen, which is why the series is so good, you can speculate indefinitely knowing that nobody will know what they will do. Maybe also I watched the season in a single day like all “normal” people do in lockdown. Incidentally I think in the next season they will use the coronavirus. suddenly a new character called Wuhan arrives on the scene…
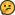
https://www.youtube.com/watch?v = OK7EePQAK8U and lc = UgyOGCDz4j1-gckk8Pt4AaABAg
*5.2. #Cuarentena*
*Álvaro Meana. Élite. Ruth Franco Apr 12, 2020*
40 views
19 subscribers
Scene of the discussion between Nano and Samuel in the series “Élite.”
Interpreted by Álvaro Meana.
The casting directors, Eva Leiraand Yolanda Serrano, have created the initiative of #Cuarentena creativa. they have proposed a series of scenes on Instagram to get everyone going in these times of lockdown. I hope you like it!
https://www.youtube.com/watch?v=8M0sW7K57Bk.

Looking at fragment 5.1, it concerns the reconstruction of the series through the characters, although they are getting into the plot. The text appears to be a comment of an analysis of the series *Money Heist*. The author has created a channel with interests in broadcasting and marketing to promote books, films, and series as multimodal forms of expression of fans, promoted from the cultural industry. In the case of this fragment, the messenger reconstructs the plot from the characters: *“I don*’*t think Alicia joins the band, it would be disappointing to lose the best enemy.”* Other plot elements are also included: “*I don*’*t think they will give away the gold and get confused with their followers. It would be too obvious and predictable,”* something which had occurred at the end of the other season. There were even explicit references to Coronavirus through the characters: *“Incidentally I think in the next season they will use the coronavirus…suddenly a new character called Wuhan arrives on the scene.”*

It is also worthwhile looking at fragment 5.2, which appears as a clear example of interactive text (Ryan et al., 2017), both with the producers and the series content. It is fully immersed in multimodal discourse. It shows how the professionals who take part in the production of Elite suggest creative tasks around this fiction for their followers. The initiative takes place through YouTube and falls without the framework of the so-called *#cuarentenacreativa*,^4^ that invites fans to offer their own productions. The fragment included presents the performance of one of the fans of the Elite series. It can be compared with many other performances or creations in the hashtag *#cuarentenacreativa*, which in turn contains 473 videos. The work relating to the fans and the professional actors would merit a separate study relating to the use of multimodal discourses between fans.

## Conclusion

This paper has analysed conversations from viewing streamed TV series. It focuses on social networks and digital media produced texts, for example, photos, magazines, and digital press. It is accepted that narrative construction is a situated process and observation is made of how it is constructed during lockdown in Spain in 2020. The daily lives of people were suddenly disrupted and everyday personal, working, and even leisure routines had to be reorganised. The streaming of television series became a relevant activity at the time, conditioned by the situation experienced. This was projected in the way in which people constructed the representations of the series as discussed on the Internet. Against this backdrop, this study proposed two objectives. Firstly, to analyse the said representations within their context and secondly, to combine quantitative and qualitative methodologies to probe into the meanings people constructed in their daily lives.

### Representations of the TV Series in Context

Talking about situated narratives, constructed from television series requires analysing the interpretations people build up and express through their conversations. This was the first objective of our analysis. Examining conversational contents led to three different thematic nuclei being outlined in context: community and institutional, interpersonal, and personal (Rogoff et al., 1993; Matusov, 2007).

We understand the context and environment in which people are immersed and which in the case of this paper is interlinked with the content of the series (Lacasa, 2020). We can speak of a triple plane on which three thematic nuclei are present. Different levels are revealed. The planes demonstrate different dimensions of the context, i.e., the awareness of the representations is represented on three interlinking planes. None of which are inseparable from the other insofar as they suggest an interpretation of the series as they appear in digital texts presented on Internet.

The first representation level involves how people become aware of an event which has institutionally affected the whole community and of which evolve over time (Jenkins, 2010). This is the awareness people have of an event that affects them collectively, but which profoundly affects other levels of their daily life as individuals, and which is apparent in the digital narratives constructed. For example, this level is shown in the thematic nucleus we have called “*The coronavirus setting*.” Considering the most frequent mentions in this nucleus, the terms most mentioned are quarantine and staying at home. These terms are present in the interpretation of the series, and this is shown through analysis of the conversations. Conversational units have been probed to conducted discourse analysis. At this level of analysis, many voices may be heard (Archakis and Tzanne, 2005). These use different pronouns to speak in the first person singular or in an impersonal way and they may, for example, appear in media texts.

The second thematic nucleus, called *“Everyday routines and social relationships,”* involves interpersonal relationships, which take place face to face, organised into a copresence which may be conducted online and offline (Bruner, 2002). In this case, the mentions centre on the said relationships, for example, alluding to the family members or friends people rely on during the pandemic. It thus alludes to the fact that the series is watched in company that implies relationships which possibly would not have come about during normal times prior to the pandemic. Relationships between family members or the need to search for a partner are mentioned through digital apps (Ryan, 2019). Again, these situations relate to series interpretation, speaking in the first person to allude to the need for social interaction. Also, people refer to specific situations in which they viewed the series. For example, when they allude to the interpersonal context, people receive the messages emitted by the series directly and personally. These are also related to routines such as mealtimes or to home entertainment situations, given that no other environment is possible.

The third thematic nucleus is on a personal level, and even the identity associated with interpretation of the series in context may be spoken of. We speak of *figured worlds* (Holland et al., 1998). In any event, this personal sphere is not removed from the collective representations of series associated with figured worlds onto which the person is projected in times of pandemic. The said worlds revolve around the concept of the series and its characters. Both are associated with individual practices by the way in which the series is viewed. People binge watch and become submerged in a world as a means of escapism which leads them to think about reconstruction of the series plot. They anticipate future activities of each character, divining the plot of the series. This is the projection of personal desires constructed within a collective framework (Page, 2012). It is also of note that at this personal level the series characters are more important than the actors who play them, because it is easier to submerge oneself in a world of fiction during the pandemic than to think of the actors, themselves, who will be experiencing the same restrictions.

In sum, the series are interpreted according to context, in keeping with their different community, interpersonal and personal levels.

### Methodological Goals

The principal methodological goal of this paper was to combine a macro and micro, quantitative, and qualitative analysis. This was the second overall objective.

The first approach to the context was undertaken through *big data analysis processes* (Panda et al., 2018). The software led to the definition of joint categories through a dual process. First, supported by analysis of the terms which most frequently appeared in the conversations—direct intervention was made by the researcher in these definitions. Second, in learning processes carried out from artificial intelligence and which led to semantically classifying the textual contributions of participants in the conversation. We are therefore dealing with categories defined from the software (Kibria et al., 2018). The combination of both types of categories received is the essential channelling of information received through social networks. Data received is structured through these categories, and we obtain quantitative data which we have partially relied upon to define the thematic nuclei mentioned through the successive processes that gave rise to an increasingly more structured information. The interaction between these data and the theory was essential for defining the before-mentioned thematic nuclei (Martínez-Borda et al., 2020).

It was necessary to use discourse analysis (Gee, 2014) to probe into the meaning people expressed in their conversations and to which no access is possible through quantitative data, at least for the moment. In this way, quantitative analysis was complemented with other types of qualitative data which was exemplified in the different examples of digital texts included through transcriptions (Lacasa, 2020). These examples were selected from among all the mentions channelled into each category and are relevant examples with regards to the objectives of the research, i.e., to analyse the dimensions present in the representations which people construct of the TV series, viewed through streaming. These analyses involve focussing on the sequences of words which make sense, and which build up a phrase. That in itself is meaningful. For example, it was relevant to analyse whether, in the internet texts, the authors of the messages speak in the first person, singular or plural, and the expressions they used to refer to context, as they expressed individual goals and feelings present in collective discourse.

### Limitations and Further Studies

The limitations of this study are challenges for the future. Firstly, this study was limited to the analysis of four streamed television series. These were chosen in keeping with the preferences of a student sample, with consideration of Spanish or foreign origin and the theme, which was either centred on youth phenomena or not. The second limitation came from the fact that this study centred on conversations in the Spanish language taking place on social media. Thirdly, data was obtained during the pandemic in Spain and not all countries had similar institutional regulations.

Future studies may be conducted along the same lines. They may broaden both the series considered and the languages and social and cultural environments from which the data is collected. It would, however, be necessary to contrast the same series or others if the weight of the context was the same in the construction of representations. Furthermore, what is increasingly more relevant is the need to progressively define the thematic nuclei and the definition categories so that the software used may be more precisely defined. The proposed categories could thus be considered a starting point to be extended and modified. Other thematic nuclei grouping could even be sought.

In summary, the pandemic has generated new online and offline universes, which have been uncommon up until now, and which also include transformations in leisure situations. Extending the study of these environments, beyond streamed TV series, is one of the pending challenges of research. Study fields and the processes of data analysis need to be extended, e.g., towards videogames or other interactive media so that narratives may be constructed collectively and in real time.

## Data Availability Statement

The original contributions presented in the study are included in the article/supplementary material, further inquiries can be directed to the corresponding author.

## Ethics Statement

Ethical approval was not provided for this study on human participants because the University of Alcalá has an Ethical Code, but not a Committee. The study has been developed according to Franzke, Aline Shakti; Bechmann, Anja, Zimmer; Michael Zimmer; Charles Ess, and the Association of Internet Researchers (2020). Internet Research: Ethical Guidelines 3.0. https://aoir.org/reports/ethics3.pdf, and the Code of Ethics for Good Research Practice. University of Alcalá, https://bit.ly/3aQ3oZT. Written informed consent for participation was not required for this study in accordance with the national legislation and the institutional requirements.

## Author Contributions

RM-B and PL conceptualised the research idea. The whole team contributed to the collection, analysis, and interpretation of data, as well as its discussion. PL redacted the study. All authors contributed to the article and approved the submitted version.

## Conflict of Interest

The authors declare that the research was conducted in the absence of any commercial or financial relationships that could be construed as a potential conflict of interest.

## Publisher’s Note

All claims expressed in this article are solely those of the authors and do not necessarily represent those of their affiliated organizations, or those of the publisher, the editors and the reviewers. Any product that may be evaluated in this article, or claim that may be made by its manufacturer, is not guaranteed or endorsed by the publisher.

## Footnotes

1 Coronavirus, covid-19, shortage, under-supplied, provision, provisioned, queues in the shop, human avalanche, waiting for them to open, lacking products, they lack products, a lot of people waiting for them to open, multitude of people waiting, Coronavirusmadrid, mob storming a supermarket, queues of people with full trolleys, CoronavirusESP, COVID19espana, people who are buying up stuff madly, under-supplied, CODVID19, pandemic, all food completely wiped out, COVID19españa, fake news, sacking, looting, coronavirusEspana, it is empty, they have hardly any, there is hardly any, multitudinous events, virus, masses of people, swept away, wiping out, covid, protocols of action, contagion, infected, people infected, contagions, people wiping out, @CoronaVid19, mask, masks, lockdown, COVID2019, provisions, buying just in case, coronavirusEspaña, INFOCoronavirus, CerradMadrid, coronaviruspanicoorealidad, bags on the head, crowds, covid_19, CoplasConCoronavirus, corona virus, covid2019, covid19, confinement, I am staying at home, mequedoencasa, yomequedoencasa, state of emergency, corona-virus, #YoMeQuedoEnCasaConCerveza, #DiarioDeCuarentena, #Yomecorono, #unidosmasfuertes.

2 To facilitate comments of textural fragments, considered as a unit of analysis, the text fragment is numbered in relation to the number of the transcription and the order it has in the transcription.

3
https://www.telemundo.com

4 #cuarentenacreativa https://www.youtube.com/hashtag/cuarentenacreativa created by Eva Leira and Yolanda Serrano, casting managers of https://leirayserrano.com an in-depth search to gain access to productions relating to the series Elite https://www.youtube.com/results?search_query=cuarentena+creativa+elite.

## References

[B1] AnsGar N. (2003). “Narratology or narratologies? Taking stock of recent developments, critique and modest proposals for future usages of the term,” in *What Is Narratology? Questions and Answers Regarding the Status of a Theory*, eds Kindt T. Müller H.-H. (Berlin: De Gruyter), 239–275.

[B2] Archakis A. Tzanne A. (2005). Narrative positioning and the construction of situated identities: evidence from conversations of a group of young people in Greece. *Narrat. Inq.* 15 267–291. 10.1075/ni.15.2.05arc 33486653

[B3] Armborst A. (2017). Thematic proximity in content analysis. *SAGE Open* 7:2158244017707797. 10.1177/2158244017707797

[B4] Barthes R. Duisit L. (1975). An introduction to the structural analysis of narrative. *New Lit. Hist.* 6 237–272. 10.2307/468419

[B5] Belson D. (2020). *¿ Qué Impacto ha Tenido la COVID-19 en las Redes de Último Kilómetro?* Reston, VA: Internet Society.

[B6] Bernhart W. Urrows D. F. (2019). *Music, Narrative and the Moving Image: Varieties of Plurimedial Interrelations.* Boston, MA: Brill Rodopi.

[B7] Boellstorff T. (2012). *Ethnography and Virtual Worlds : A Handbook of Method.* Princeton, NJ: Princeton University Press.

[B8] Boyd D. (2008). “Why youth love social network sites: the role of networked publics in teenage social life,” in *Youth, Identity, and Digital Media*, ed. Buckingham D. (Cambridge, MA: MIT Press), 119–142.

[B9] Bruner J. (1986). *Actual Minds, Possible Words.* Cambridge, MA: Harvard University Press.

[B10] Bruner J. (2002). *Making Stories: Law, Literature, Life.* Cambridge, MA: Harvard University Press.

[B11] Chung D. S. Yoo C. Y. (2008). Audience motivations for using interactive features: distinguishing use of different types of interactivity on an online newspaper. *Mass Commun. Soc.* 11 375–397. 10.1080/15205430701791048

[B12] Combes C. Glevarec H. (2020). Differentiation of series and tastes for TV series: the French case. *Media Cult. Soc.* 43 860–885. 10.1177/0163443720977277

[B13] Demuth C. Raudaskoski P. Raudaskoski S. (2020). Editorial: lived culture and psychology: sharedness and normativity as discursive, embodied and affective engagements with the world in social interaction [Editorial]. *Front. Psychol.* 11:437. 10.3389/fpsyg.2020.00437 32218763 PMC7078356

[B14] Dencik L. (2020). Mobilizing media studies in an age of datafication. *Telev. New Media* 21 568–573. 10.1177/1527476420918848

[B15] Ensslin A. (2011). “From (W)reader to breather: cybertextual de-intentionalization and Kate Pullinger’s breathing wall,” in *New Narratives*, Kindle Edn. eds Page R. Thomas B. (Lincoln, NE: University of Nebraska Press).

[B16] Fiske J. (1987). *Television Culture*, 2nd Edn. Abingdon: Routledge.

[B17] Gee J. P. (2014). *An Introduction to Discourse Analysis: Theory and Method*, 4th Edn. London: Routledge.

[B18] Genette G. (1980). *Narrative Discourse (1972)*, trans. J. E. Lewin (Oxford: Blackwell).

[B19] Greimas A. J. Collins F. Perron P. (1990). *Narrative Semiotics and Cognitive Discourses.* London: Pinter.

[B20] Grimm K. Jacobucci R. McArdle J. J. (2017). *Big Data Methods and Psychological Science. Psychological Science Agenda.* Available online at: https://bit.ly/3DmU0Z4 (accessed April 6, 2021).

[B21] Hall S. (1997). *Representation: Cultural Representations and Signifying Practices.* London: Sage in association with the Open University.

[B22] Hemphill L. Kocurek C. A. Rao X. (2018). “Approaches to understanding identity, gamers, fans, and research methods,” in *The Routledge Companion to Media Fandom*, eds Scott S. Click M. A. (Abingdon: Routledge), 45–54.

[B23] Holland D. Lachicotte W. Skinner D. Cain C. (1998). *Identity and Agency in Cultural Worlds.* Cambridge, MA: Harvard University Press.

[B24] Jenkins H. (2010). Transmedia storytelling and entertainment: an annotated syllabus. *Continuum* 24 943–958. 10.1080/10304312.2010.510599

[B25] Jenkins H. Clinton K. Purushotma R. Robison A. J. Weigel M. (2006). *Confronting the ChaIIenges of Participatory Culture: Media Education for the 21 Century.* Chicago, IL: MacArthur Foundation.

[B26] Jenkins H. Lashley M. C. Creech B. (2017). A forum on digital storytelling| interview with Henry Jenkins. *Int. J. Commun.* 11:8.

[B27] Jewitt C. Bezemer J. Van Leeuwen T. (2020). Tribute to Gunther Kress (1940–2019): reflecting on visuals that shaped his work. *Vis. Commun.* 19 3–11. 10.1177/1470357219883517

[B28] Jewitt C. Bezemer J. J. O’Halloran K. L. (2016). *Introducing Multimodality.* London: Routledge.

[B29] Jones R. H. Chik A. Hafner C. A. (2015). *Discourse and Digital Practices: Doing Discourse Analysis in the Digital Age.* New York, NY: Routledge, Taylor & Francis Group.

[B30] Kavoori A. Lashey M. C. Creech B. (2017). A forum for digital storytelling| voices for a new vernacular: a forum on digital storytelling — introduction. *Int. J. Commun.* 11 1057–1060.

[B31] Kibria M. G. Nguyen K. Villardi G. P. Zhao O. Ishizu K. Kojima F. (2018). Big data analytics, machine learning, and artificial intelligence in next-generation wireless networks. *IEEE Access* 6 32328–32338. 10.1109/ACCESS.2018.2837692

[B32] Kitchin R. (2014). Big data, new epistemologies and paradigm shifts. *Big Data Soc.* 1:2053951714528481. 10.1177/2053951714528481

[B33] Kress G. (2012). “Multimodal discourse analysis,” in *The Routledge Handbook of Discourse Analysis*, eds Gee J. P. Handford M. (New York, NY: Routledge), 35–50.

[B34] Kress G. Van Leeuwen T. (2006). *Reading Images The Grammar of Visual Design*, 2nd Edn. London: Routledge.

[B35] Lacasa P. (2020). *Adolescent Fans: Practices, Discourses, and Communities.* New York, NY: Peter Lang. 10.3726/b14291

[B36] Larrain A. Haye A. (2019). Self as an aesthetic effect [Conceptual Analysis]. *Front. Psychol.* 10:1433. 10.3389/fpsyg.2019.01433 31316422 PMC6611353

[B37] Martínez-Borda R. Lacasa P. Castillo H. (2020). Big and small data: watching and discussing television series on streaming. *Cuad.Info Comun. Medios Ibero Am.* 49 331–335. 10.7764/cdi.49.27297

[B38] Matusov E. (2007). In search of ‘the appropriate’ unit of analysis for sociocultural research. *Cult. Psychol.* 13 307–333. 10.1177/1354067X07079887

[B39] Mittell J. (2015). *Complex TV: The Poetics of Contemporary Television Storytelling.* New York, NY: New York University Press.

[B40] Monfort N. (2007). “Narrative and digital media,” in *The Cambridge Companion to Narrative*, ed. Herman D. (Cambridge: Cambridge University Press), 172–186. 10.1017/CCOL0521856965.012

[B41] Murray J. Lashley M. C. Creech B. (2017). A forum on digital storytelling| interview with Janet Murray. *Int. J. Commun.* 11 1078–1080.

[B42] Nærland T. U. (2018). Fictional entertainment and public connection: audiences and the everyday use of TV-series. *Telev. New Media* 20 651–669. 10.1177/1527476418796484

[B43] Negra D. (2020). Pandemic television. *Film Crit.* 44 23–25. 10.3998/fc.13761232.0044.407 26400043

[B44] Ong J. C. Negra D. (2020). The media (Studies) of the pandemic moment: introduction to the 20th anniversary issue. *Telev. New Media* 21 555–561. 10.1177/1527476420934127

[B45] Page R. Thomas B. (2011). “Introduction,” in *New Narratives*, eds Page R. Thomas B. (Lincoln, NE: University of Nebraska Press), 1–16. 10.2307/j.ctt1df4h49.5

[B46] Page R. E. (2010). *New Perspectives on Narrative and Multimodality.* New York, NY: Routledge.

[B47] Page R. E. (2012). *Stories and Social Media: Identities and Interaction.* New York, NY: Routledge.

[B48] Panda M. Abraham A. Hassanien A. E. (2018). *Big Data Analytics: A Social Network Approach.* Boca Raton, FL: Taylor & Francis Group.

[B49] Paoletti J. Bisbey T. M. Zajac S. Waller M. J. Salas E. (2021). Looking to the middle of the qualitative-quantitative spectrum for integrated mixed methods. *Small Group Res.* 52 641–675. 10.1177/1046496421992433

[B50] Papacharissi Z. Lashley M. C. Creech B. (2017). A forum on digital storytelling| interview with ZiziPapacharissi. *Int. J. Commun.* 11 1069–1073.

[B51] Parks L. (2020). Field mapping: what is the “Media” of media studies? *Telev. New Media* 21 642–649. 10.1177/1527476420919701

[B52] Passalacqua F. Pianzola F. (2011). Defining transmedia narrative: problems and questions. Dialogue with Mary-Laure Ryan. *Enthymema* 4 65–71. 10.13130/2037-2426/1188

[B53] Piñón J. (2019). Disruption and continuity on telenovela with the surge of a new hybrid prime-time fictional serial: the super series. *Crit. Stud. Telev.* 14 204–221. 10.1177/1749602019838885

[B54] Recuber T. (2017). “Digital discourse analysis: finding meaning in small online spaces,” in *Digital Sociology in Everyday Life*, eds Daniels J. Gregory K. (Bristol: Policy Press), 963–1270.

[B55] Rogoff B. Mistry J. Goncu A. Mosier C. (1993). Introduction. The concepts of guided participation and cultural universals and variation. *Monogr. Soc. Res. Child Dev.* 58 1–18. 10.1111/j.1540-5834.1993.tb00433.x 8284000

[B56] Rudrum D. (2005). From narrative representation to narrative use: towards the limits of definition. *Narrative* 13 195–204. 10.1353/nar.2005.0013 34409987

[B57] Ryan M.-L. (1991). *Possible Worlds, Artificial Intelligence and Narrative Theory.* Bloomington, IN: Indiana University Press.

[B58] Ryan M.-L. (2005). “On the theoretical foundations of transmedial narratology,” in *Narratology Beyond Literary Criticism: Mediality, Disciplinarity*, eds Meister J. C. Kindt T. Schernus W. (Berlin: Walter de Gruyter), 1–24. 10.1515/9783110201840

[B59] Ryan M.-L. (2006). From parallel universes to possible worlds: ontological pluralism in physics, narratology, and narrative. *Poetics Today* 27 633–674.

[B60] Ryan M.-L. (2007). “Toward a definition of narrative,” in *The Cambridge Companion to Narrative*, ed. Herman D. (Cambridge: Cambridge University Press), 22–36. 10.1017/CCOL0521856965

[B61] Ryan M.-L. (2010). Narratology and cognitive science: a problematic relation. *Style* 44 469–495. 10.1002/wcs.1305 26005512 PMC4441006

[B62] Ryan M.-L. (2011). “The interactive onion. Layers of user participation in digital narrative texts,” in *New Narratives*, eds Page R. Thomas B. (Lincoln, NE: University of Nebraska Press), 35–62. 10.2307/j.ctt1df4h49.7

[B63] Ryan M.-L. (2015). *Narrative as Virtual Reality 2: Revisiting Immersion and Interactivity in Literature and Electronic Media*, 2nd Edn. Baltimore, MD: Johns Hopkins University Press.

[B64] Ryan M.-L. (2016). Transmedia narratology and transmedia storytelling. *Artnodes* 18 1–10. 10.7238/a.v0i18.3049

[B65] Ryan M.-L. (2019). “Narration in various media,” in *The Living Handbook of Narratology*, eds Hühn P. (Hamburg University). Available online at: http://www.lhn.uni-hamburg.de/article/narration-various-media (accessed April 6, 2021).

[B66] Ryan M.-L. Lashley M. C. Creech B. (2017). *A Forum on Digital Storytelling— Interview with Marie-Laure Ryan [Digital Storytelling]*, Vol. 11. Available online at: http://ijoc.org/index.php/ijoc/article/view/6780/1960

[B67] Schaeffer J.-M. (2010). “Le traitementcognitif de la narration,” in *Narratologies Contemporaines :Approchesnouvelles Pour la Theìorie et L’analyse du Reìcit*, eds Pier J. Berthelot F. (Paris: Archives contemporaines), 215–232.

[B68] Serafini F. Reid S. F. (2019). Multimodal content analysis: expanding analytical approaches to content analysis. *Vis. Commun.* 10.1177/1470357219864133

[B69] Sloan L. Quan-Haase A. (2017). “Introduction to the handbook of social media research methods: goals, challenges and innovations,” in *The SAGE Handbook of Social Media Research Methods*, eds Sloan L. Quan-Haase A. (Los Angeles: SAGE), 4–11. 10.4135/9781473983847

[B70] Stein L. E. (2015). *Millennial Fandom: Television Audiences in the Transmedia Age.* Iowa City, IA: University of Iowa Press.

[B71] Stein N. Trabasso T. (1981). “What’s in a story? An approach to comprehension and instruction,” in *Advances in Instructional Psychology*, Vol. 2 ed. Glaser R. (Hillsdale, NJ: Lawrence Erlbaum), 213–268.

[B72] Sundet V. S. Peteresen L. N. (2020). Ins and outs of transmedia fandom: motives for entering and exiting the SKAM fan community online. *Poetics* 84:101510. 10.1016/j.poetic.2020.101510

[B73] Tannen D. Hamilton H. E. Schiffrin D. (2015). *The Handbook of Discourse Analysis*, 2nd Edn. New York, NY: John Wiley & Sons, Inc.

[B74] Thon J.-N. I (2016). *Transmedial Narratology and Contemporary Media Culture.* Lincoln, NE: University of Nebraska Press.

[B75] Thornborrow J. (2015). *The Discourse of Public Participation Media: From Talk Show to Twitter.* Abingdon: Routledge.

[B76] Thurlow C. Dürscheid C. Dieìmoz F. (2020). *Visualizing Digital Discourse: Interactional, Institutional and Ideological Perspectives.* Berlin: De Gruyter Mouton.

[B77] Werron T. Ringel L. (2020). Pandemic practices, part one. how to turn “Living through the COVID-19 pandemic” into a heuristic tool for sociological theorizing. *Sociologica* 14 55–72. 10.6092/issn.1971-8853/11172

[B78] Woo S. E. Tay L. Proctor R. W. (2020). *Big Data in Psychological Research.* Washington, DC: American Psychological Association.

[B79] Zamith R. Lewis S. C. (2015). Content analysis and the algorithmic coder: what computational social science means for traditional modes of media analysis. *Ann. Am. Acad. Pol. Soc. Sci.* 659 307–318. 10.1177/0002716215570576

[B80] Zikopoulos P. (2012). *Understanding Big Data: Analytics for Enterprise Class Hadoop and Streaming Data.* New York, NY: McGraw-Hill.

